# Antiangiogenic Therapies and Extracranial Metastasis in Glioblastoma: A Case Report and Review of the Literature

**DOI:** 10.1155/2015/431819

**Published:** 2015-06-23

**Authors:** Mohamed H. Khattab, Ariel E. Marciscano, Simon S. Lo, Michael Lim, John J. Laterra, Lawrence R. Kleinberg, Kristin J. Redmond

**Affiliations:** ^1^Department of Radiation Oncology & Molecular Radiation Sciences, Johns Hopkins University, 401 North Broadway, Suite 1440, Baltimore, MD 21231-5678, USA; ^2^Department of Radiation Oncology, Case Western Reserve University, University Hospitals Seidman Cancer Center, Cleveland, OH 44106, USA; ^3^Department of Neurosurgery, Johns Hopkins University, 600 N. Wolfe Street, Phipps Room 123, Baltimore, MD 21287, USA; ^4^Department of Neurology/Neuro-Oncology, Johns Hopkins University, 601 N. Caroline Street, Baltimore, MD 21287, USA

## Abstract

We present a case report of a patient with glioblastoma multiforme (GBM) complicated by extracranial metastasis (ECM) whose survival of nearly four years surpassed the anticipated life expectancy given numerous negative prognostic factors including EGFRvIII-mutation, unmethylated MGMT promoter status, and ECM. Interestingly, while this patient suffered from locally aggressive disease with multiple intracranial recurrences, the proximal cause of death was progressive extracranial disease and complications related to pulmonary metastases. Herein, we review potential mechanisms of ECM with an emphasis upon glioblastoma molecular and genetic profiles and the potential implications of targeted agents such as bevacizumab.

## 1. Introduction

Glioblastoma multiforme (GBM) is one of the most lethal human malignancies. The median survival of patients with newly diagnosed GBM treated with adjuvant radiation therapy (RT) with concurrent and adjuvant temozolomide (TMZ) remains dismal at 14.6 months. The majority of patients ultimately succumb to local recurrence in the central nervous system [1]. Extracranial metastasis (ECM) is a rare phenomenon of GBM, estimated to occur in less than 2% of diagnoses [2, 3]. However, a recent study from Europe reported that circulating glioblastoma cells were present in 20.6% of GBM patients [4].

Current literature suggests that the median time from initial diagnosis to detection of ECM is approximately 8.5 months and the interval of time between detection of distant metastasis and death is only 1.5 months, suggesting that development of ECM is a harbinger of morbidity and mortality [2, 3]. The factors that predispose GBM patients to the development ECM are currently not well understood.

## 2. Case Presentation

**Figure 1:**
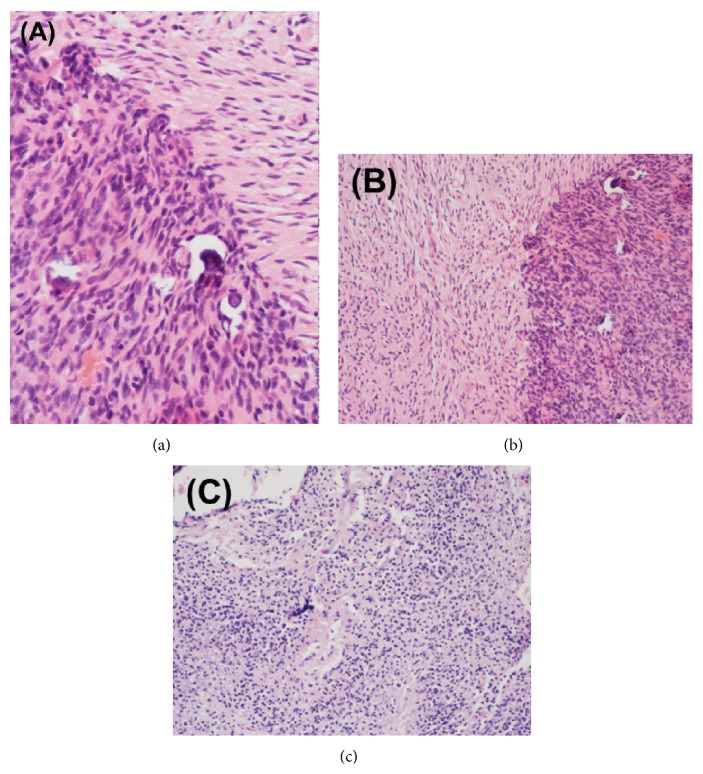
Surgical pathological specimens from (a) initial craniotomy and (b-c) repeat craniotomy for recurrent intracranial disease. Histopathological analysis of the initial (a) right temporal lobe specimen demonstrated glioblastoma with small cell features. The pathological specimen after second resection (b) demonstrated active glioblastoma, almost entirely viable with <5% necrosis. Following the third and final (c) craniotomy for locally recurrent disease, predominantly viable (approximately 15% necrosis) active glioblastoma was identified. Molecular studies were negative for MGMT methylation and EGFR amplification was detected by FISH.

A previously healthy 51-year-old right-handed African American gentleman initially presented following an unwitnessed seizure. MRI revealed focal enhancement in the posterior temporal lobe measuring approximately 9 mm as well as T2 hyperintensity within the right insular cortex and right temporal lobe. He was empirically treated for HSV encephalitis until CSF HSV PCR assay returned negative. Given persistent mental status changes, MR imaging at 4 and 9 months after initial presentation revealed an enhancing temporoparietal mass with interval enlargement between scans. He underwent right temporal craniotomy with postoperative pathology consistent with WHO grade IV astrocytoma (GBM) with small cell features (Figure 1(a)). Specific histopathological findings of cellular atypia with variable morphology ranging from packed cells with relatively homogeneous ovoid nuclei to less cellular areas with a whorled configuration, foci of necrosis with scattered calcifications, numerous mitoses, and endothelial proliferation were noted. Immunohistochemical staining demonstrated strong staining for vimentin and negative staining for EMA. Additional tumor analysis revealed an unmethylated MGMT gene promoter and EGFR amplification, later confirmed to be EGFRvIII positive.

He was treated with adjuvant radiotherapy (RT) delivered (6000 cGy in 30 fractions) with concurrent daily temozolomide (TMZ, 75 mg/m^2^/day) followed by adjuvant TMZ (150 mg/m^2^/day × 5 days every 28 days). Follow-up MRI after two cycles of adjuvant TMZ revealed increased enhancement within the surgical cavity. TMZ was switched to metronomic scheduling (120 mg daily) during the third cycle due to concern of progression versus pseudoprogression for a total of 8 additional cycles. MR imaging a month later revealed marked progressive enhancement and the patient underwent repeat resection with postoperative imaging indicating gross total resection and pathologic confirmation of recurrent GBM (Figure 1(b)).

At 27 months following his initial diagnosis, the patient initiated bevacizumab monotherapy which was discontinued after four cycles due to radiographic progression with development of a mildly enhancing intracavitary nodule. A third craniotomy was performed with pathology demonstrating mitotically active, recurrent GBM (Figure 1(c)). The patient did well postoperatively without radiographic evidence of disease. He was clinically stable without neurologic deficits and maintained an excellent performance status at this time. Surveillance MRI two months following surgery demonstrated nodular tumor recurrence with dural extension and he enrolled in a clinical trial investigating genetically modified T-cells expressing anti-EGFRvIII. The protocol consisted of apheresis, followed by cyclophosphamide, fludarabine, T-cell infusion, and eight infusions of IL-2 (this trial is registered with Clinical Trial: NCT01454596).

After two months of therapy in the aforementioned clinical trial he developed left homonymous hemianopia with brain MRI revealing infiltrative enhancement around the right temporal lobe surgical cavity and increased vasogenic edema throughout the right temporal and parietal lobes. He underwent reirradiation (3600 cGy in 20 fractions) with concurrent bevacizumab. He continued with adjuvant bevacizumab for an additional two cycles and maintained a good performance status throughout therapy.

**Figure 2:**
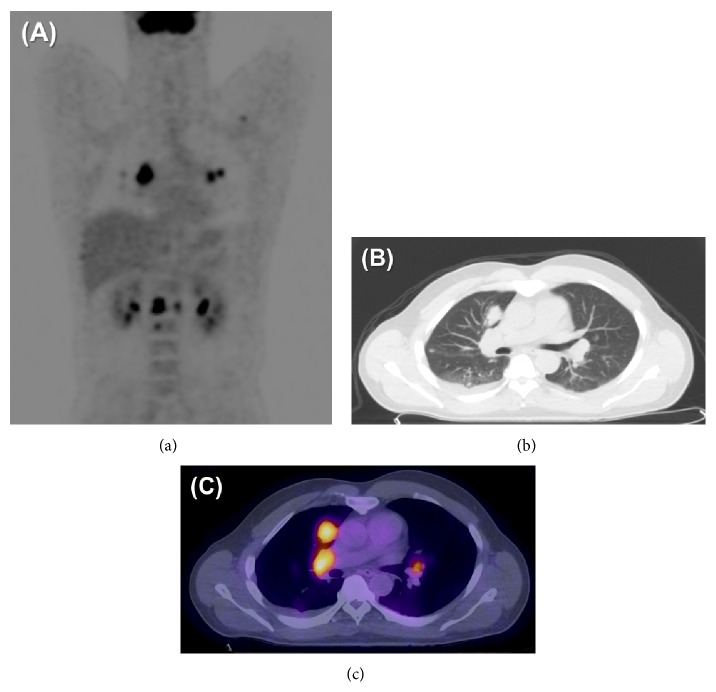
(a) Whole body FDG PET imaging demonstrating hypermetabolic osseous and visceral metastases including bilateral pulmonary nodules and hilar adenopathy. (b) CT imaging demonstrating mediastinal adenopathy, multiple pulmonary nodules, and right pleural effusion. (c) Coregistered PET/CT imaging demonstrating intense FDG uptake within GBM lung metastases.

The patient developed back pain and abdominal discomfort approximately 40 months following initial diagnosis and 3 months following reinitiation of bevacizumab therapy. CT imaging of the chest demonstrated multiple pulmonary nodules, including a 2.5 cm right middle lobe nodule and a 1.2 cm left lower lobe nodule along with bilateral hilar adenopathy. A staging fluorodeoxyglucose positron emission tomography (FDG PET) scan noted abnormal uptake within several right hilar lymph nodes and diffuse osseous FDG uptake within the sternum, thoracic, lumbar, and pelvic bones. The pulmonary nodules identified on CT imaging were also found to be FDG-avid, right middle lobe (SUV 11.5), two left upper lobe nodules (SUV 6.4 and 5.1), and right upper lobe (SUV 5.0) (Figure 2). Flexible bronchoscopy with biopsy was performed and demonstrated malignant spindle cell tumors with geographic necrosis consistent with metastatic GBM. The tumor cells were positive for GFAP, weakly positive for S100, and negative for synaptophysin, EMA, HHV-8, HMB-45, desmin, CD34, keratin, chromogranin, and CD117. MIB-1 proliferative index was approximately 40%. AFB and GMS stains were negative.

**Figure 3:**
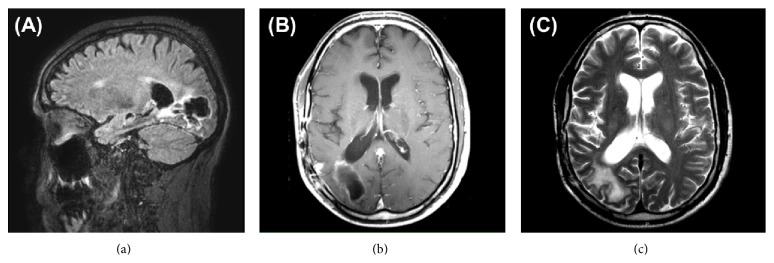
Intracranial imaging during final course of salvage therapy with bevacizumab and carboplatin. (a) FLAIR, (b) T1-weighted axial, and (c) T2-weighted axial magnetic resonance imaging demonstrating stable intracranial disease at time of respiratory decline. Postsurgical hyperintensity surrounds area of resection in the right temporal and occipital lobes communicating with the right occipital horn, unchanged with respect to prior imaging.

One month later, he resumed treatment with bevacizumab and, in the 43rd month, carboplatin. Restaging FDG PET imaging after 2 cycles revealed extracranial progression of disease with worsening pulmonary and skeletal metastases. Of note, synchronous brain MRI was stable at this time with no evidence of intracranial recurrence or progression (Figure 3). He continued with a third and final cycle of concurrent bevacizumab and carboplatin with continued decline of his respiratory status. 46 months from initial diagnosis, he ultimately passed away secondary to acute respiratory failure. At the time of his death, there was no clinical or radiographic evidence of disease within the central nervous system.

## 3. Discussion

It is currently unknown if the use of bevacizumab is implicated in the potentiation of ECM. It is notable that extracranial dissemination of this patient's disease coincided with administration of bevacizumab therapy.

Concerns have been raised that anti-VEGF therapies, including bevacizumab, lead to transformation of primary and recurrent GBM into a more infiltrative phenotype [5, 6]. The withdrawal and reversal of VEGF inhibition have been observed to rapidly increase regrowth of tumor vasculature [7] and therapies administered after bevacizumab failure often only provide transient tumor control [8]. Potential mechanisms have recently been elucidated, illustrating that anti-VEGF inhibitors alter tumor programming and growth pattern of recurrent GBMs in a fashion that promotes resistance to antiangiogenic therapies as well as a more invasive phenotype [5]. It is also postulated that glioblastoma stem cells may differentiate directly into new vessels in the presence of VEGF blockade, yielding a conduit for additional tumor growth and dissemination [9].

The rationale for bevacizumab use in glioblastoma is that inhibition of VEGF normalizes tumor vasculature, thus decreasing tumor interstitial pressure. This improves access to chemotherapeutic drugs as well as oxygen delivery, thus improving efficacy of radiation therapy [10–12]. However, it has also been argued that normalization of the blood-brain barrier, as shown by other antiangiogenic agents when combined with TMZ, may yield less tumor kill, thus generating debate [10, 13].

It remains unknown if extracranial dissemination is impacted by the interaction of targeted treatments and biological factors such as MGMT methylation status or EGFR amplification [14]. A landmark study by Hegi et al. reported that GBM patients with methylated MGMT promoters derive significant benefit from the addition of adjuvant TMZ to RT in comparison to their unmethylated counterparts. Furthermore, MGMT promoter methylation was found to be an independent favorable prognostic factor [15].

Extrapolation from other disease sites may suggest a potential relationship between MGMT status and ECM [16–19]. Kohonen-Corish et al. reported that MGMT hypermethylation is present in up to one-third of patients with distant melanoma metastases and there is considerable heterogeneity of MGMT expression depending on the site of metastasis. Additionally, MGMT promoter methylation did correlate with resistance to TMZ therapy, reemphasizing the role of MGMT in modulating chemosensitivity [20]. With regard to extracranial metastasis among other primary brain tumors, MGMT promoter hypermethylation has been associated with ECM in oligodendrogliomas [16].

EGFR gene amplification is another biological factor potentially implicated in ECM of primary brain tumors [21]. EGFR gene amplification has been observed in pediatric low-grade gliomas that disseminate possibly through a mechanism of induced FABP7 nuclear translocation [16]. FABP7 is involved in glial-guided neuronal migration and EGFR-mediated nuclear translocation may be associated with a propensity for dissemination [22]; however, this relationship has yet to be established in GBM.

As we enter an era of emerging novel therapeutics including immunotherapies and targeted agents, there is potential for unforeseen side effects and unintended detrimental sequelae as a consequence of treatment. While unmethylated MGMT promoter status and amplified EGFR expression, as seen in our case report above, predict a poor prognosis, the relation of these biomarkers to success of targeted antiangiogenic therapies warrants further investigation.
